# Fatal snake bites – sociodemography, latency pattern of injuries

**DOI:** 10.1186/1745-6673-8-7

**Published:** 2013-03-25

**Authors:** Chidananda PS Rao, Parameshwar Shivappa, Veeresh R Mothi

**Affiliations:** 1 Department of Forensic Medicine and Toxicology, Shimoga Institute of Medical Sciences, Sagar Road, Shimoga, Karnataka, 577201, India; 2 Department of Internal Medicine, Shimoga Institute of Medical Sciences, Shimoga, RGUHS, Karnataka, India; 3 Department of Forensic Medicine and Toxicology, Shimoga Institute of Medical Sciences, Shimoga, RGUHS, Karnataka, India

**Keywords:** Snake bite, Agriculturist, Poverty, Bite-admission time, Fang marks

## Abstract

**Background:**

India is a thickly populated country; apart from having biodiversity among people, climate does change from place to place. Western Ghats of South India harbors variety of plantations and diverse creatures. Agriculture is the primary occupation of the people and some tribes living in these regions. Here majority are callous/ ignorant in employing neither advanced farming techniques nor safety precautions, hence are exposed to bites and stings by animals. Of these, snake bites cause significant mortality and morbidity. Proper care for some of these individuals is out of reach. Identification of offending snake, snake bite injury or findings of envenomation is a key not only for the administration of antisnake venom but also for the victim to realize that he needs an expert care. Unless he believes it to be a critical snake bite and not a thorn prick, scorpion sting or a spider bite he will not approach a health care provider. To know about these dangerous signs that may help the victim to realize it as a case of snake bite, current study is employed on fatal cases in this region.

**Methods:**

60 fatal snakebite cases were studied retrospectively for 5 years with an objective to know the socio-demography, latency and pattern of injuries in rural Southern India.

**Results:**

Most of the victims were males, in the age group of 31-50 years and were at risk of snake bites while farming. Large sample of subjects approached traditional therapists and were deprived of essential care in the critical first few hours after snake bite. Fang marks (90%), local ecchymoses (50%) and internal hemorrhage (28.3%), were the frequent demonstrable signs appreciated at autopsy.

**Conclusion:**

Snakebite is a neglected, endemic, occupational (farming) disease of the poor and there is need for National Snakebite Prevention Programme for curtailing this menace.

## Background

Indian physicians were known from 326 BC for their ability to treat poisonous snakebites. Having known about venomous snakes for more than 2500 years [1], we still fall back short in curtailing snakebite-induced deaths. There are 52 species of venomous snakes in India [2], of which 24 are considered as most important [3]. Incidence and frequency of snake bite vary in different geographic regions, depending on several factors like climate, ecology, biodiversity, distribution of snakes & human density [4]. India is thought to have more snakebites than any other country [1]. In 2005, it caused 45,900 deaths (1% of deaths from all causes) with an age standardized death rate of 5.4 per one lakh population for India [1]. Treatment is primarily aimed at neutralizing the venom containing an array of bioactive protein molecules with specific antivenin. Unless the signs of bite or signs of envenomation are recognized, victim may not visit a health care provider and there is a strong association between snakebite-induced mortality with poverty, mistaken identity, mismanagement by untrained village based traditional therapists, poor transportation facilities, delay at arrival to medical centres and improper dosing of Antisnake venom [1,5]. Current data available to this region are solely based on, under reporting hospital statistics [1,6] and, less effort has been made to evaluate socio-demographic factors, bite-admission time, and Snake identification by victims and factors which expose humans to risks of bite. Objectives of this study were aimed at determining the neglected factors and, to assess the pattern of injuries, that to, the incidence of fang marks in fatal bites.

## Methods

This retrospective study was conducted on fatal snake bite victims, subjected for medico-legal autopsy at Govt. Dist. Mc Gann hospital, Shimoga, from 2005 to 2010.

Information about victim (age, gender, occupation and socioeconomic status; Above poverty line or Below poverty line – Based on State Govt. laid 15 criteria including Tax payment, annual income of Rs. 12,000), the snake bite (Identification of snake, time of snake bite, place of incident), its management (Pre-hospital care, latency between bite and admission) and pattern of injuries (Type of injury, number, site, physical signs of envenomations), were sought by going through postmortem reports, hospital case sheets, based on ration cards issued by govt. revenue department, inquest reports and police case dairies. Edema was graded as Grade I, if it was localised, as II, if extended one joint above/ below the site of bite and as III, if extensive (Whole limb).

## Results

During this study period, 60 cases of fatal snake bites amounting to 2.29% (60 of 2625) of unnatural deaths were subjected for medico-legal autopsy at our centre. Majority were poor, male agriculturists, exposed to snakes while working at fields. Socio-demographic profile of the victims is depicted in Table 1 and no case of fatal snake bite was reported in the months of January and February.

**Table 1:** Socio-demography of fatal snake bites victims

**Parameter**	**Number (n = 60)**	**Percentage**
***Age group in years***		
0-10	1	1.7
11-20	9	15
21-30	3	5
31-40	13	21.7
41-50	17	28.3
51-60	11	18.3
> 60	6	10
***Gender***		
Male	35	58.3
Female	25	41.7
***Occupation***		
Agriculturist	46	76.7
House wife	3	5
Students/Unemployed	11	18.3
***Socio-economic status***		
Below poverty line	52	86.7
Above poverty line	8	13.3
***Time of incident***		
4 am – 8 am	6	10
8 am – 12noon	20	33.3
12noon – 4 pm	27	45
4 pm – 8 pm	6	10
8 pm – 4 am	1	1.7
***Place of the incident***		
Fields	44	73.3
Residence	12	20
Garden	3	5
Public place (Bus stand)	1	1.7
***Seasonal trend***		
Summer (March – June)	21	35
Rainy season (July – Oct)	24	40
Winter (Nov – Feb)	15	25

**Figure 1:**
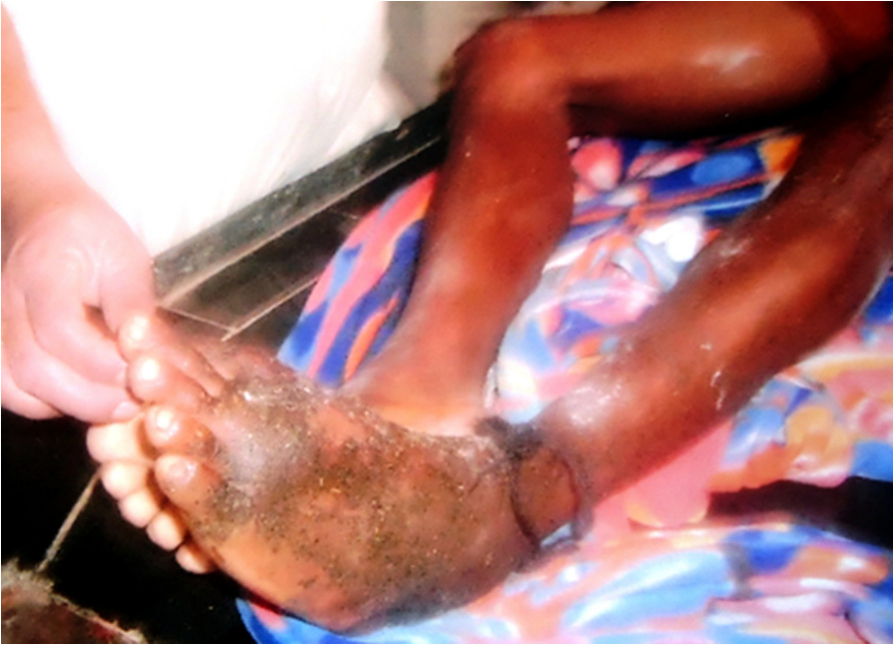
Photograph showing application of plant extracts at the bite site by a traditional therapist.

Majority did not seek medical aid in the crucial first hour following bite and proper pre-hospital care was sought only in 30% of cases and most of them approached an untrained traditional therapist, who followed crude procedures in the forms of putting local incisions, suctioning, application of plant extracts (Figure 1), snake stones etc. Treatment employed and bite to admission time is depicted in Table 2.

**Table 2:** Characteristics of therapy provided to victims and bite-admission time

**Parameter**	**Number (n = 60)**	**Percentage**
***Medication sought by untrained local therapist/ Quack***		
Yes	42	70
No	18	30
***Snake identification***		
Identified as Cobra	9	15
Identified as Viper	3	5
Unidentified	48	80
***Proper pre-hospital care***		
Not employed	42	70
Employed	18	30
***Bite-admission time/ Latency***		
< 1 hour	4	6.7
1-2 hour	30	50
2-4 hour	18	30
4-12 hour	2	3.3
>12 hr	6	10

**Figure 2:**
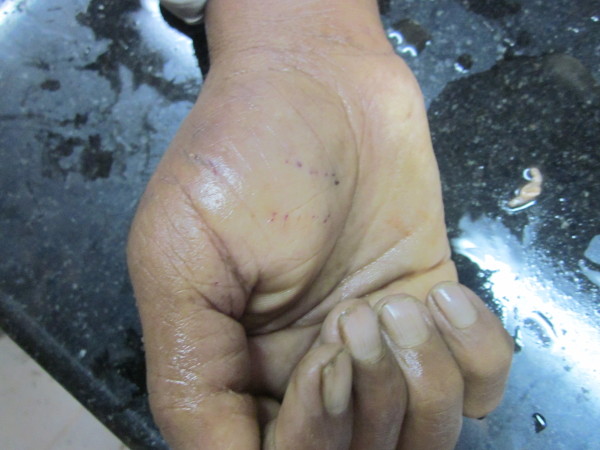
Photograph showing complete snake bite mark.

**Figure 3:**
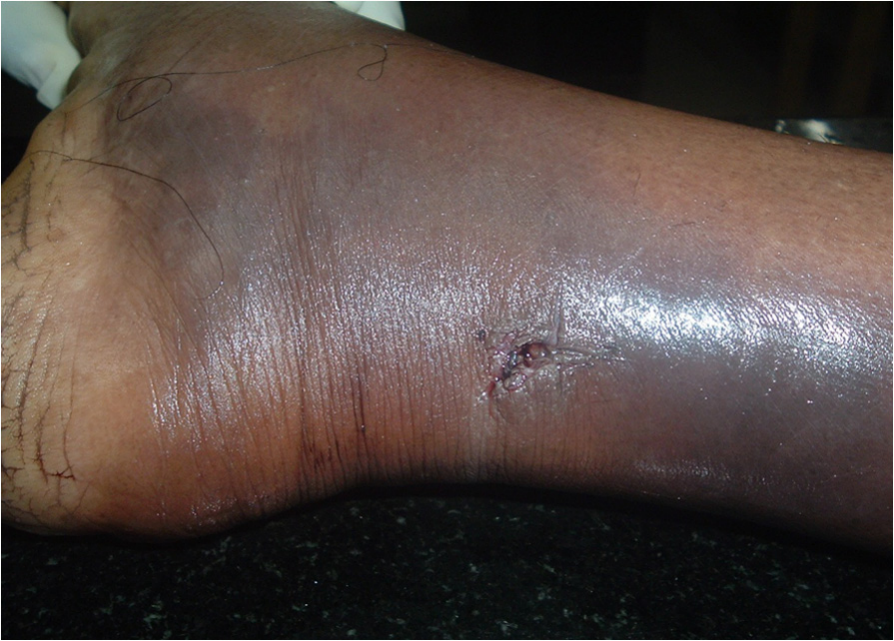
Photograph showing application of Cruciate Incision at the site of bite.

Pattern of injuries ranged from Scratches to punctured wounds and simple darkening of skin at the site of bite (ecchymoses) and, the same with duration of survival are depicted in Table 3. Fang marks appreciated varied from one set (65%) to multiple sets (10%) and sometimes whole teeth bite mark (Figure 2) was visible. Distance between the fang marks ranged from 0.2 cm to 2.5 cm with a mean of 1.2 cm. Hemorrhagic manifestations observed includes local bleeds (50%), intra-cerebral hematomas (1.7%), Epicardial (1.7%), intra-abdominal (1.7%), mesenteric (5%), retroperitoneal bleeds (10%) and blood extravasations into muscle plains (8.3%), frequently into iliopsoas. Unscientific methods were employed by medical officers at rural health centres in the form of incision (Figure 3), vacuum application and cautery in 15% of cases.

**Table 3:** Pattern of injuries noted in fatal bites

**Parameter**	**Number (n = 60)**	**Percentage**
***Site of bite***		
Hands	18	30
Feet	27	45
Forearm	6	10
Leg	8	13.3
Trunk	1	1.7
Head & Neck	Nil	-
***Pattern of injuries/ signs***		
Scratches	12	20
***Fang marks***		
Solitary	6	10
One set	39	65
Multiple sets	9	15
Absent	6	10
Edema		
Grade I	23	38.3
Grade II	9	15
Grade III	9	15
Ecchymoses/ Darkening at the site of bite	30	50
Oozing at bite site	6	10
Toxic blisters	8	13.3
Cellulitis	8	13.3
Internal hemorrhage	17	28.3
***Duration of survival***		
< 1 hour	10	16.7
1-3 hour	12	20
3-6 hour	13	21.7
6-12 hour	12	20
12-24 hour	6	10
1-2 days	1	1.7
2-7 days	5	8.2
>7 days	1	1.7

## Discussion

From our literature search, it is felt that, there appears to be presence of only fragmentary epidemiological data on a preventable accidental disease/ death and its public health importance has been systematically underestimated and, all together, it has joined the group of neglected diseases.

When we corroborated the literature from various countries, similar higher incidence of snake bites in agricultural [7] male workers [1,7,8], aged 21 to 50 years [7,8], more so at day time [6], in rainy and summer months [6-8] was found. In developed countries most of snakebites occur on recreational activities, whereas in developing countries it is more of an occupational disease [9]. Persons working in agricultural fields [6] are at risk of being bitten by snakes, as they neither employ advanced irrigation methods, nor take safety measures like wearing gloves, boots etc., either because of unaffordability or they are too uncomfortable to wear them in hot, humid conditions. Rate was equally higher in rainy and warmer months and this could be due to flooding of the holes, termite mounds in monsoon and heating of the ground in summer, forcing the snakes to encroach and habitat out into the surroundings of human dwellings. Added to this, grains stored in houses attract rats, in turn creating a food chain, increasing the chances of snake-human contacts.

Relationship of poverty v/s snakebite incidence and mortality has been clearly demonstrated [5]. The survival of many of the rural poor, in India and other developing regions of the world, like Africa [10,11], Latin America [12] and other parts of Asia [6,13], is dependent upon their non-mechanized, low-cost farming or tilling techniques in rainy seasons and it is an irony that it is exactly these practices that place them at such high risk of snakebite to their feet, legs and hands [6,7,14,15]. There is a strong correlation between global ‘Per capita Government Expenditure on Health and snake bite mortality’ and the tragedy is highest mortality is in those countries which have limited resources for buying antivenom [5].

More than 2/3^rd^ of the victims approached therapy centres with an unknown bite or with a bite from an unidentified species; this could be due to ignorance or poor visibility [8,9,15], this is in contrast to the study at Nepal where 60% of the times victims were able to identify the snakes [6].

Most cases were from rural areas and they did not seek medical aid in the crucial first hour following bite with an average delay of 2.5 hours, and the causes for delay were: their ignorance, callous attitude towards the dangerousness of the situation, indulgence in practice of unscientific methods of therapy and lack of transportation facilities [6,14]. Proper pre-hospital care was sought only in 30% of cases [14] and the rest approached an untrained traditional therapist, who employed unscientific procedures in the forms of putting local incisions, suctioning, application of plant extracts, mud, garlic, snake stones etc.

Lower limbs were the frequent sites of bite, followed by upper limbs, that, to feet and hands [7,14,15]. Fang marks were appreciable in 90% of cases. Number and distance between puncture marks varied with a median of 1.05 ± 0.68 cm, and is probably dependent on, the size of the snake, angle of strike, number of strikes and on the presence of protective clothing/ covering.

Bleeding manifestations following viperine bites are common but are not limited to it. It occurred as local ecchymoses to systemic bleeds in the form of retroperitoneal, intra-cerebral, inter and intra muscular bleeds [16]. Bleeding and edema at the site of bite is due to the local action of venom (proteases, phospholipases, vasculotoxins and autacoids), inducing increased vascular permeability [17]. Systemic bleeding is due to platelet deficiency and the co-existing defibrination syndrome [18]. Cerebral vascular impairments after snake bite is rare, Mosquera [19] has reported cerebral vascular complications in 2.6%, mostly hemorrhagic and rarely ischemic in his series of 309 snake bite . Intravenous inoculation of venom triggers rapid onset of and extensive coagulation disorders [16]. Toxic blisters seen at the site of bite in 13.3% of cases could be due to tissue necrotic effects of the venom.

Duration of survival ranged from less than an hour to more than a week (Median = 5 hrs) and 78% succumbed within 12 hours following bite, and this depends on the type of snake, size of the snake, age, site of bite, amount of venom injected, type of pre-hospital care sought and latency between bite and hospitalisation. Fatality among our subjects occurred much earlier than the average described for cobras and sea snakes as 5–15 hours [20] and for vipers as up to 48 hours following bite [21].

## Conclusion

•Snakebite is a neglected, life threatening emergency demanding immediate antivenom.

•It is disease of poverty, endemic to farming regions.

•Syndromic/ Protocol approach for management of unknown snake bite, recognising snake bites into three groups [22] – Painful progressive swelling, Progressive weakness and Bleeding syndrome; appears logical in reducing mortality.

•There is a need for enactment of National programme on snake bite prevention; aiming at improving quality of care, health education, financial aid to curtail non-mechanised farming techniques and steps for implementation of WHO guidelines on Snakebite management for South East Asia Region.

### Ethical approval

Study is done in accordance with Guidelines on Bio-Medical Research on Human participants (2006) prescribed by Indian Council of Medical Research, which is in compliance with the Helsinki Declaration. As per the guidelines this retrospective study doesn’t require Institutional Ethical Committee Clearance. However as the study was done on fatal cases brought for medico-legal autopsy with requisition from the investigating agency and Executive magistrates, consent and approval has been obtained on a case to case basis by the legal guardians.

## Competing interests

The authors declare that they have no competing interests.

## Authors’ contributions

CP participated in acquisition of data, literature search and carried out analysis, interpretation, and drafting the manuscript. PS treated some victims; did acquisition of data and participated in drafting and revising the manuscript. VM supervised and carried out revising the manuscript. All authors read and approved the article.
